# Semi-robotic 6 degree of freedom positioning for intracranial high precision radiotherapy; first phantom and clinical results

**DOI:** 10.1186/1748-717X-5-42

**Published:** 2010-05-26

**Authors:** Jürgen Wilbert, Matthias Guckenberger, Bülent Polat, Otto Sauer, Michael Vogele, Michael Flentje, Reinhart A Sweeney

**Affiliations:** 1 Department of Radiation Oncology, University Hospital Würzburg, Josef-Schneider-Str. 11, 97080 Würzburg, Germany

## Abstract

**Background:**

To introduce a novel method of patient positioning for high precision intracranial radiotherapy.

**Methods:**

An infrared(IR)-array, reproducibly attached to the patient via a vacuum-mouthpiece(vMP) and connected to the table via a 6 degree-of-freedom(DoF) mechanical arm serves as positioning and fixation system. After IR-based manual prepositioning to rough treatment position and fixation of the mechanical arm, a cone-beam CT(CBCT) is performed. A robotic 6 DoF treatment couch (HexaPOD™) then automatically corrects all remaining translations and rotations. This absolute position of infrared markers at the first fraction acts as reference for the following fractions where patients are manually prepositioned to within ± 2 mm and ± 2° of this IR reference position prior to final HexaPOD-based correction; consequently CBCT imaging is only required once at the first treatment fraction.

The preclinical feasibility and attainable repositioning accuracy of this method was evaluated on a phantom and human volunteers as was the clinical efficacy on 7 pilot study patients.

**Results:**

Phantom and volunteer manual IR-based prepositioning to within ± 2 mm and ± 2° in 6DoF was possible within a mean(± SD) of 90 ± 31 and 56 ± 22 seconds respectively. Mean phantom translational and rotational precision after 6 DoF corrections by the HexaPOD was 0.2 ± 0.2 mm and 0.7 ± 0.8° respectively. For the actual patient collective, the mean 3D vector for inter-treatment repositioning accuracy (n = 102) was 1.6 ± 0.8 mm while intra-fraction movement (n = 110) was 0.6 ± 0.4 mm.

**Conclusions:**

This novel semi-automatic 6DoF IR-based system has been shown to compare favourably with existing non-invasive intracranial repeat fixation systems with respect to handling, reproducibility and, more importantly, intra-fraction rigidity. Some advantages are full cranial positioning flexibility for single and fractionated IGRT treatments and possibly increased patient comfort.

## Background

In the last decade, there have been major technological advances, of note cone-beam CT (CBCT) [1-3], 3D fluoroscopy [4-6] and 6 degrees of freedom (DoF) treatment couches [7-10], all commercially available and in clinical use. These have made not only submillimeter but also sub-degree positioning possible, allowing reduction of safety margins and also giving clinicians the confidence to perform even radiosurgical procedures without invasive fixation, using for example thermoplastic masks [11,12]. Without IGRT, such masks allow repositioning accuracy of about ± 2 mm (SD) and about ± 2° [13,14]. The IGRT process relativises this inaccuracy somewhat, however, image acquisition and position correction, even with 6DoF remote couches takes time and judging from our experiences, the required corrections exceed the capabilities of the HexaPOD to correct remotely on average every third fraction (unpublished data, RS, MG). In such cases, manual pre-corrections need to be performed with the base couch. Large rotational corrections can in turn themselves induce translational anatomical changes inside a thermoplastic mask [15] which may be critical, so even with IGRT and 6DoF couches, repositioning accuracy is still important; less is always better, especially for rotational errors. Some may argue that rotational errors are not an issue, but especially for larger irregular volumes or multiple tumors treated simultaneously [16] ignoring rotations may reduce coverage or increase organ at risk exposure. Finally, intra-fractional patient motion, especially for radiosurgical procedures is of utmost importance and not negligible in thermoplastic masks [17,18].

In this work, we describe the system, pre-clinical and pilot-patient results of a novel concept, combining 4 well known and clinically proven systems to maximize their individual high potential, namely the vacuum mouthpiece (vMP), 6 DoF couch, CBCT, infrared(IR). The novelty is the manual IR-based prepositioning of the head to within ± 2 mm and ± 2° before allowing a robotic, 6DoF treatment couch to complete the remaining required rotations and translations to within the system accuracy of 0.1 mm and 0.1°. We thus hypothesize previously unattained accuracy in all 6 DoF with high reliability and speed, while possibly being more flexible and patient friendly than other repeat fixation aides. This can be achieved with minimal radiation dose to the patient, as ionizing verification could in principle be necessary only once during the entire course of fractionated radiotherapy.

## Proposed clinical procedure (Figure 1)

**Figure 1:**
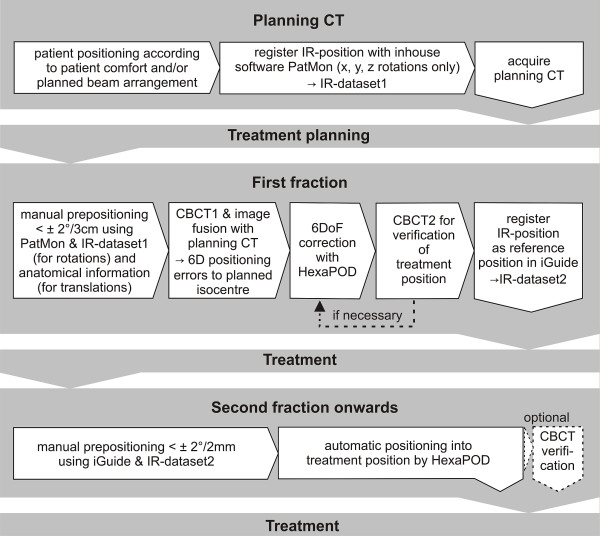
**Workflow from planning CT to second treatment**. Should there be no IR-cameras in the planning CT room, an additional CBCT would be necessary at the first fraction (dotted line). Abbreviations: IR = Infrared, CBCT = cone-beam CT, DoF = degrees of freedom.

The position of the cranium is defined in the planning CT. In contrast to all current fixation systems, this position is not predefined or limited by some rigid (non-) invasive structure of sorts (e.g. mask systems, stereotactic rings systems). The initial reference structure is the 3D volume of the head itself. At first treatment, CBCT and image fusion is used for verification of the correct patient position and this geometric position of the cranium is saved via an IR frame, which is connected to the vMP. From the second fraction onwards, positioning occurs only according to this isocentre-specific IR-position. A more detailed description is given in the following section.

## Materials

### Infrared array- based reproducible positioning and fixation

**Figure 2:**
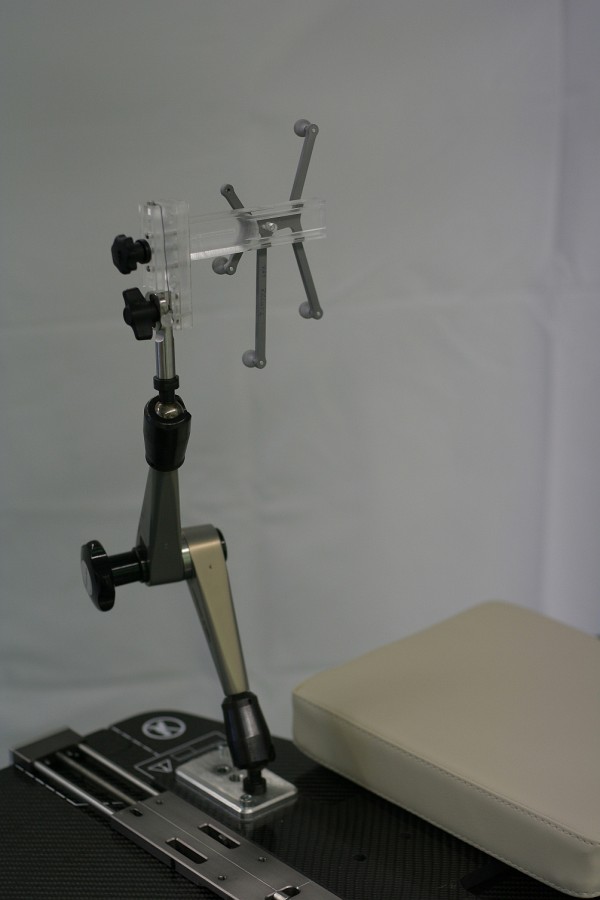
**Infrared-Mechanical Arm unit**. An infrared reference frame is connected to the mechanical arm which in turn is connected to the treatment table via a self-centering bracket. Before patient positioning, the arm and IR-frame are rotated out of the way as shown so the patient can lie down on the headrest.

**Figure 3:**
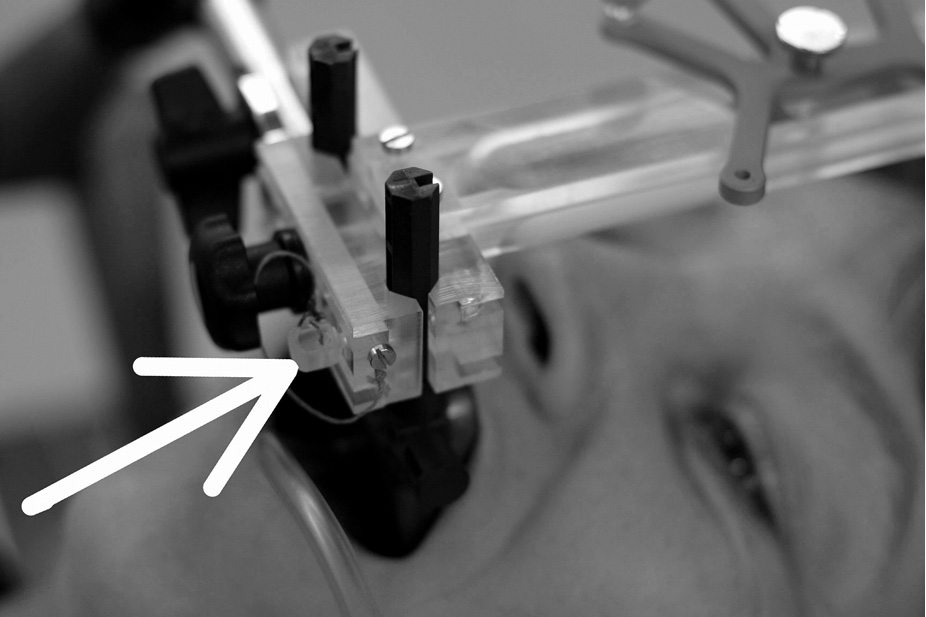
**Fixated subject**. The infrared frame has been reproducibly connected to the anterior arms of the vMP. The safety pin (arrow) will only slide through the respective hole in the anterior arm if the connection is correct. (The shown subject has provided written consent for the publication of this image).

The central element and the only patient specific hardware is the vMP(Medical Intelligence GmbH, Schwabmünchen, Germany). Its production has been previously described[19,20]. In short, an individual dental/upper palate impression with a small vacuum area against the upper palate is made using a quickly hardening vinyl-poly-siloxane material. Production takes 5-10 minutes. Using a vacuum pump, air can be evacuated through the underside of the mouthpiece from this vacuum-area. allowing objectively consistent connection of the vMP to the patient's upper dentition. The connection of the vMP to the treatment table is achieved via a mechanical arm which allows full 6 DoF movement until locked by turning a screw (ATLAS MultifunctionalARM™, Medical Intelligence GmbH, Schwabmünchen, Germany). This mechanical arm is attached to a base-plate which itself is attached to the treatment table with one self-centering clamp. A reference frame with an array of four infrared markers is rigidly attached to the mechanical arm (Figure 2). Once the patient is positioned on the treatment table with vMP in place and vacuum verified, this mechanical arm-reference frame unit is reproducibly clamped onto the anterior arms of the vMP (Figure 3).

No individualized headrest is required; a standard headrest serves well for strictly supine position. However, for rotated positioning of the head, a flat pillow (Figure 2) or an individualized vacuum cushion is recommended. Ideally, the headrest or cushion is not fixated to the base-plate. This "floating" headrest allows the repositioning process to rely solely on the vMP/IR-frame connection, maximizing the concept of tensionless fixation.

**Figure 4:**
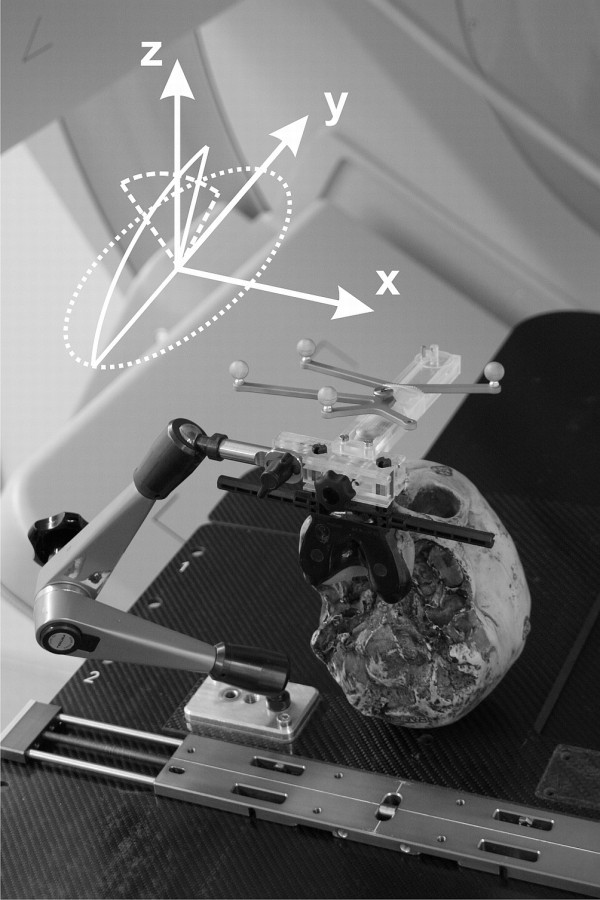
**Phantom positioning**. The anthropomorphic phantom fixated in treatment position. The achievable rotations under IR guidance using this IR-frame are illustrated as is the room coordinate system (x, y, z).

All other materials (CBCT, ceiling mounted infrared cameras and 6 DoF treatment couch (HexaPOD with *iGuide*-Software (Version 1.0), Medical Intelligence GmbH, Schwabmünchen Germany)) are commercially available in the scope of the Access Linac (Elekta, Crawley, UK). Ideally, an identical infrared camera (Polaris, NDI) is mounted in the planning CT room so that the initial patient position can be transferred to the treatment room. In-house software ("PatMon" [10]) was used for this purpose in this study. The room coordinates are defined as x (left-right), y (cranio-caudal) and z (anterior-posterior) with respect to a supine patient on the treatment couch (Figure 4).

## Methods

### Planning CT

The patient lies down comfortably on the table in a standard head mould and inserts the vMP. Correct seat of the vMP is verified when the manometer on the vacuum pump shows values in the range of -0.3 to -0.6 mbar. Then the IR-reference array, rigidly connected to the mechanical arm on the base plate (Figure 2), is attached to the vMP anterior arms. A safety pin, which ensures reproducibility of the connection IR-frame to vMP, must be applied (Figure 3).

No special attention is required to align the head to lasers, nor is there a need for reference markings.

The head is then manually positioned as required, then fixated by tightening the screw on the mechanical arm. Patients can be positioned with any pitch, roll or yaw rotation of the head offering additional degrees of freedom for treatment planning or improved patient comfort. The position of the infrared markers, as read by the ceiling mounted infrared cameras is saved within the PatMon software (= *IR-dataset1*).

After the planning CT, the mechanical arm is unlocked, the safety pin pulled and the reference frame pulled off the vMP anterior arms thus releasing the patient.

Treatment planning can be performed as usual.

Treatment plan data, the vMP and the *IR-dataset1 *are transferred to the treatment unit.

### First treatment

**Figure 5:**
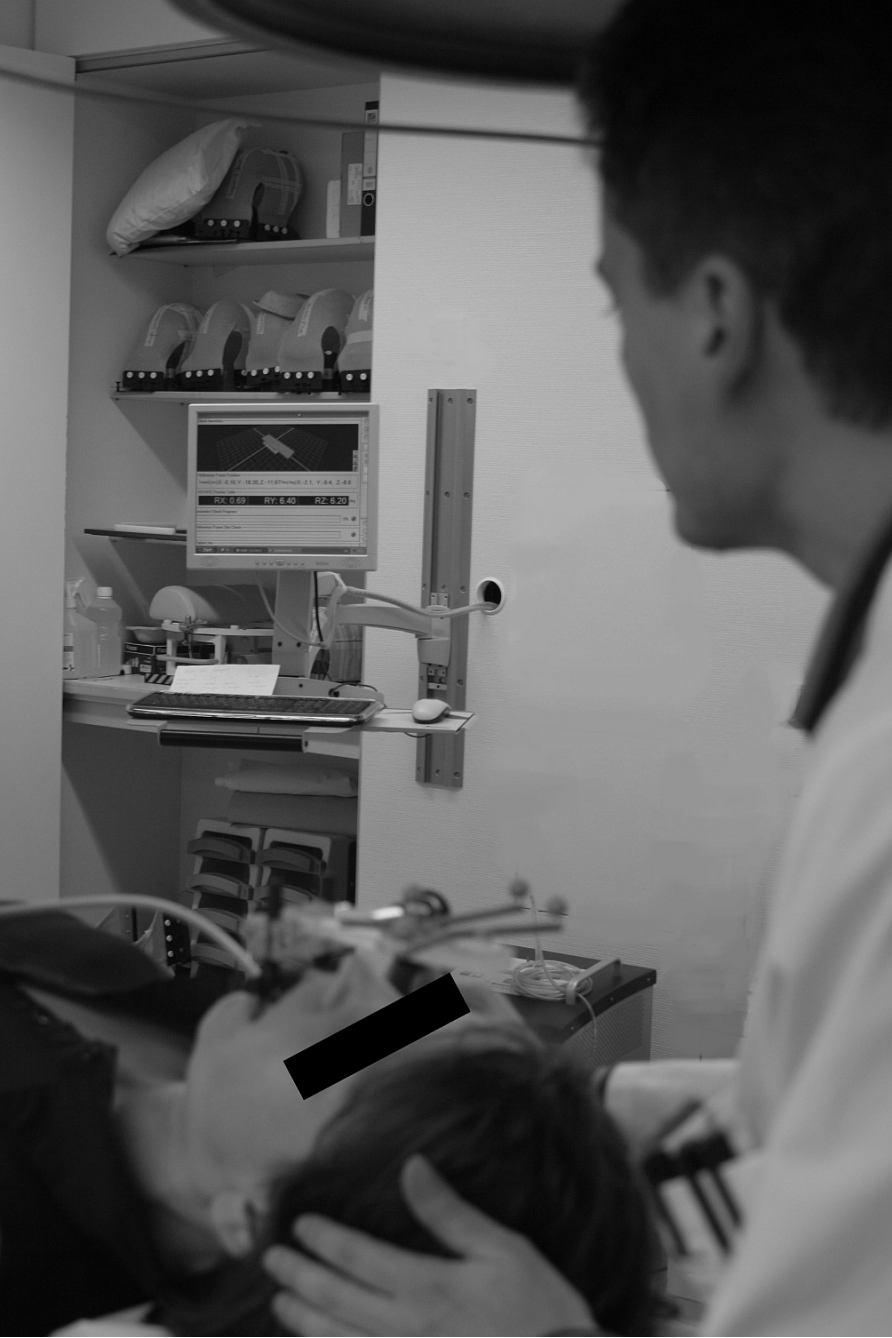
**Manual prepositioning**. A subjects head is rotated around all 3 room axes to < ± 2°. Note how the head is manipulated with one hand, the mechanical arm with the other hand. The required infrared-based translations and rotations are read off the treatment room iGuide screen, visible in the background.

After reminding the patient not to be surprised should slight table rotations be felt, the vMP is applied to the patient, the patient's head positioned on the head rest and the IR-frame/mechanical arm unit is attached to the vMP, this connection again verified by the safety pin. Standing at the cranial end of the patient, the therapist now manually rotates the head into the reference position from the planning CT to within ± 2° around all axes using the respective *IR-dataset1 *from the planning CT (Figure 5); the required rotations are displayed on an in-room computer monitor. This ensures that the rotational inaccuracy is reduced to within the capabilities of the HexaPOD. At this point, the mechanical arm is locked by turning the screw. Now translations can be executed using the base couch so that the laser isocentre roughly corresponds to treatment isocentre (tumor) position. This position can usually be approximated to within ± 3 cm in all translatory axes.

A CBCT is performed; the volume data set is matched to the planning CT images using the automatic grey value algorithm. The alignment clipbox is generally defined to encompass the entire skull. The resulting required translational and rotational positioning shifts to align to isocentre in 6 DoF are corrected remotely with the HexaPOD itself; should the required corrections however exceed HexaPOD capability, then rough approximation with the base couch must precede the HexaPOD movement.

We recommend repeating the CBCT as verification prior to treatment as this patient and isocentre specific IR position stored within *iGuide *will be the reference position for all following fractions (= *IR-dataset2*).

After treatment, the mechanical arm is unlocked; the vMP is removed, rinsed with water and stored in a patient specific box for the next treatment.

### From second treatment onwards

The patient is pre-positioned to within ± 2 mm and ± 2° manually as described, however this time using the *IR-dataset2 *reference position from the first treatment. Attention must always be paid to verifying correct vMP position (audible hiss should the vacuum against the upper palate break, and visible on the manometer gauge) prior to and during this manual prepositioning. After again locking the mechanical arm, the HexaPOD should automatically complete the rest of the IR- based positioning to submillimetric and tenth degree precision.

## Phantom Study

### 1.) Attainable repositioning accuracy of the reference frame onto the vMP

This system critically depends on the repositioning accuracy of the IR-reference frame onto the mouthpiece, tested by repositioning the mechanical arm/IR-frame unit onto the anterior arms of the vMP 20 times. The vMP remained rigidly attached to a cranial anthropomorphic phantom which itself was screwed against the base-plate (Figure 4). The 6 DoF infrared deviations from the baseline position were noted.

### 2.) Range of rotations detectable by the IR system

One of the inherent advantages of this method is that, at least theoretically, the head can be fixated in a tilted position, ± 90° around the x, y and z axis. This freedom is however limited not only by anatomy, but also by the IR-frame geometry. To determine the actual registration range of the IR-frame by the cameras, the phantom was rotated from a supine (0°) position around all axes and the maximally registered angle was noted.

### 3.) Attainable phantom results

The entire clinical procedure as described above was tested using the abovementioned phantom, at this point however not fixated against the base plate. The vMP however remained rigidly attached to the phantom; Planning CT slice thickness was 2 mm. Three users (one radiation therapist, one physicist and one physician, all naive to 6DoF manual prepositioning) each positioned the phantom 10 times, totaling 30 repositionings, including the initial position according to the planning CT infrared information (*IR-dataset1*).

To determine the feasibility of the manual prepositioning according to infrared information, the time from beginning the manual pre-positioning to reaching the required ± 2° and ± 2 mm was noted.

After CBCT1 and image registration to the planning CT using the clipbox surrounding the skull and the "grey value" algorithm, the required translatory and rotational corrections were noted. The duration of the ensuing HexaPOD correction of these values was also measured. After another CBCT(2) and image registration to planning CT dataset, the final deviations from isocentre were noted, again in 6 DoF.

### 4.) Subject study

To evaluate the manual prepositioning process on humans, an individual vMP was made for 5 informed and consenting adult volunteers. Time was measured from the beginning of the manual positioning process (lowest base table level, vMP inserted but mechanical arm loose, head turned about 30° to one side), up to when the subjects were positioned under infrared guidance to within ± 2 mm and ± 2° of an initially saved supine baseline infrared-position. This was repeated 5 times each by 5 different "therapists" (n = 30), all with little to no experience in manual 6 DoF, IR-based positioning.

### 5.) Pilot study

Between March and July 2008, 7 patients scheduled for fractionated intracranial radiotherapy at our department were included in this study on a prospective protocol after providing written informed consent. All were treated according to the described method, with a CBCT performed after positioning according to IR (CBCT1) and after each fraction (CBCT2). An additional verification CBCT (CBCT1v) was made after the HexaPOD corrections at first treatment prior to saving that IR position as reference for the following fractions. Thus, the CBCT1 values showed the accuracy of the entire semi-automatic IR-based repositioning process (manual prepositioning + HexaPOD corrections). Intra-fraction movement was calculated by subtraction of the CBCT1 values from CBCT2 values.

To determine positioning- and intra-fraction duration, the time was measured from when the patient entered the treatment room up to the beginning of CBCT1 and CBCT2 acquisitions, respectively.

Deviations are reported as described by van Herk [21]; for each patient, the mean (systematic error) and standard deviation (SD; random error) of all deviations during treatment were calculated. The group mean error (M) is defined as the average of all systematic errors; Σ is defined as the SD of the systematic errors. The root-mean-square of the random errors was calculated as σ. Deviations in all 3 translational and rotational axes were calculated separately as was the length of the 3D translational vector. Safety margins for compensation of rigid setup errors and intra-fraction errors were calculated using the formula 2.5Σ + 0.7σ.

## Results

### 1.) Attainable repositioning accuracy of the reference frame onto the vMP

Repositioning the IR frame 20 times showed a standard deviation of frame position of ≤ 0.1 mm and ≤ 0.1° around all axes. No translation or deviation was > ± 0.1 mm or degree, demonstrating that repositioning accuracy of the IR frame onto the vMP is possible to at least the resolution of the IR system itself (Table 1).

**Table 1:** Infrared frame repositioning results.

	X(mm)	Y(mm)	Z(mm)	rot x (°)	rot y (°)	rot z (°)
	
SD	0.04	0.01	0.05	0.028	0.022	0.015
max	0.1	0.05	0.1	0.1	0.05	0.05

Standard deviation (SD) and maximum repositioning deviations when repeatedly connecting the frame to a fixated mouthpiece on the phantom.

### 2.) Range of rotations detectable by the IR system

Using the 4-Arm infrared-array as seen on Figures 3, 4 and 5, only rotations around the z axis could be measured around 360°. Detection of rotations around the x axis was limited to -19° (chin away from chest) and +90° (chin towards chest). Detection of rotations around the y-axis was limited to about ± 40°.

### 3.) Attainable phantom results

Prepositioning the phantom manually to within ± 2° according to IR parameters (n = 30) took 91 ± 31 seconds (mean ± SD). This manual prepositioning was performed to within a root mean square error of 1.8 ± 2.5 mm and 0.58 ± 0.46° respectively.

Correction of these values by the HexaPOD took 21 ± 4.1 seconds (mean ± SD).

Table 2 shows the final positioning (deviation of CBCT2 to planning CT) in the individual directions or axes. Averaged over all translations (xyz) and rotations, a root mean square error of 0.2 ± 0.2 mm and 0.07 ± 0.08° was reached respectively. The mean 3D vector was 0.4 ± 0.2 mm.

**Table 2:** Final deviations of phantom position compared to planning CT after 6DoF correction with HexaPOD.

	**X(mm)**	**Y(mm)**	**Z(mm)**	**3D-vector (mm)**	**rot x (°)**	**rot y(°)**	**rot z(°)**
	
M	0.0 ± 0.1	0.1 ± 0.3	0.1 ± 0.2		0.01 ± 0.1	0.06 ± 0.1	0.06 ± 0.1
***σ ***(Mean ± SD)	0.1 ± 0.1	0.3 ± 0.2	0.2 ± 0.1	0.4 ± 0.2	0.08 ± 0.1	0.08 ± 0.1	0.06 ± 0.1

grey value match of CBCT2 with planning CT

group mean error (M)

root mean square of random errors (σ)

Degree of freedom (DoF)

### 4.) Subject study

Repositioning humans to within ± 2 mm and ± 2° (n = 30) took 56 ± 22 seconds (mean ± SD). Interuser variance was small. However, a steep learning curve was obvious (mean initial positioning time was 182 seconds (range 92-243 seconds). Also, it was found that manual prepositioning is best performed by guiding the head with one hand while simultaneously guiding the mechanical arm close to the mouthpiece with the other hand (Figure 5).

### 5.) Pilot Patient Study

Specific patient information is listed in Table 3. In total, 110 complete datasets of 117 fractions (94%) were available for evaluation (229 CBCT datasets). All 110 fractions could be evaluated for intra-fraction errors. Due to the different procedure at the initial fraction, only 102 inter-fraction displacements were included in the analysis.

**Table 3:** Pilot Patient data.

	Age	**K.S**.	BMI (kg/m^2^)	Diagnosis	Dental Status	Fx. treated	% Fx. imaged before treatment	% Fx. imaged after treatment	CBCT2 repeat due to > 1.5 mm/° error
1	58	80	29	Brain metastasis breast cancer	full	13	69	69	1
2	55	80	23	Brain metastasis SCLC	full	13	100	100	0
3	42	80	25	Brain metastasis breast cancer	full	10	90	90	0
4	46	70	20	Brain metastasis breast cancer	no teeth	10	100	100	0
5 *	69	90	27	Pituitary adenoma	3 teeth	29	97	100	7
6^+^	65	80	26	Glioblastoma	full	31	97	97	2
7	46	70	30	Clival metastasis NSCLC	full	11	100	83	0

* positioned chin to chest; ^+ ^painful occipital scar thus oblique position

K.S. = Karnofsky Score

Fx. = Fractions

min. = minutes

7 fractions (6%) could not be evaluated due to CBCT downtime, during which verification was done by orthogonal portal images.

Individual translational and rotational deviations are shown in Table 4.

**Table 4:** Inter-fraction errors.

	Translations (mm)			Rotations (°)		
	
	X	Y	Z	x	y	z
M	0	0.6	0	0.02	0.06	0.18
Σ	0.7	0.8	0.5	0.02	0.06	0.18
σ	0.8	0.1	0.7	0.26	0.28	0.44

Results were obtained from registration of planning CT-with cone-beam CT (CBCT1), based on the cranium as region of interest, using grey value matching. group mean error(M), standard deviation(SD) of systematic errors(Σ), root-mean-square of random errors(σ)

The 3D displacement vector after IR based semi-robotic patient positioning was 1.6 ± 0.8 mm (mean ± SD) and the maximum 3D Vector was 3.8 mm. Margins ranging from 1.7 mm in AP to 2.3 mm in lateral direction were calculated for compensation of these setup errors.

In a total of 7 fractions, the initial IR-based position was corrected a second time by the HexaPod after CBCT1 because the deviation was > 2 mm. 6 of these were performed on Patient 5 who was initially positioned with chin to chest, an obviously uncomfortable/unphysiologic position, resulting in rotations >1.5° around the lateral axis (x) in 7 of 28 fractions. Excluding this patient from data analysis however did not alter the 3D vector results, only the mean rotations around the x-axis were reduced from 0.37 to 0.26°.

Mean patient preparation and positioning time (from entering room to CBCT1) was 4.5 ± 1.5 minutes.

Mean total treatment time (from entering room to CBCT2) was 15.03 ± 6.01 minutes.

Intra-fraction movement results of all 110 evaluable cases are shown in Table 5. The mean 3D Vector of intra-fraction movement was 0.6 ± 0.4 mm. Calculation of required margins to account for intra-fraction movement gave submillimetric values (maximum 0.8 mm).

**Table 5:** Intra- fraction movement.

	Translations (mm)			Rotations (°)		
	
	X	Y	Z	x	y	z
M	-0.1	0	0	0	-0.06	-0.15
Σ	0.2	0.2	0.1	0.09	0.15	0.24
σ	0.4	0.4	0.2	0.15	0.23	0.31
max	1.8	1.1	1.4	0.8	1.1	2

Results were obtained from registration of planning CT-with cone-beam CT (CBCT2), based on the cranium as region of interest, using grey value matching.

## Discussion

Currently, the most common fixation systems for fractionated stereotactic radiotherapy are based on thermoplastic masks; these use the entire skull as reference structure, which is fairly ambiguous due to its circular form. Only the nasal ridge and orbital rims act as a landmark; however, these structures are covered by skin, itself non-rigid and susceptible to swelling or shrinkage. Thus, the only easily accessible rigid reference structure for cranial purposes is the upper jaw, ideally equipped with more than 2 or 3 teeth. Following this logic, a variety of mouthpiece-based systems have been described [19,20,22]. Nonetheless, these are not as reliably precise as expected due to the imbalance of positioning a fairly large mass such as the head relative to a small reference structure as the mouthpiece. Any tension or torsional forces exerted on the mouthpiece would cause slight but noticeable deviations [23].

It is hard to improve on the excellent results attainable with thermoplastic masks using IGRT; their suboptimal repositioning accuracy can be compensated by correcting all translations prior to treatment and, if the respective couch is available, also rotations around all axes. However, some of the still existing limitations of thermoplastic masks can be overcome using the presented method, namely

a) Usually, rotations > 1.5° can't be corrected by 6DoF treatment couches alone requiring approximation of the required coordinates by base couch manipulation. This is no exception, an analysis on thermoplastic mask series in our department showed this to be necessary in about 30% of fractions (unpublished data RS&MG). All 8 (7%) residual rotations >1.5° in this pilot study occurred in the patient who was originally positioned in an uncomfortable position, again emphasizing the importance of tensionless fixation, an issue even for invasive frames [24]. Thus, using a system as precise as this one correctly, that is initially positioning the patients in a comfortable position in the planning CT, should allow the manual pre-positioning process to reliably reduce the remaining translations and rotations to ranges easily attainable by a 6DoF treatment table such as the HexaPOD.

b) Allowing the fixation system to adapt to the patient instead of forcing the patient into a supine position. Up to a certain degree, the mechanical arm allows tilted head positions should these be more comfortable for the patient or required for planning reasons. The extent of tilt is currently limited by the fiducial array recognition of the IR-cameras (Figure 4). Such positioning flexibility may be especially useful in particle therapy where ideally, the distance from nozzle to patient surface is minimal. At least theoretically it could also be used as an alternative to expensive ion/proton beam gantries in particle therapy [25].

c) This system is fully independent of intra-fraction facial contour changes (i.e. cortisone induced swelling or tumor induced cachexia.

d) Tolerance problems of claustrophobic patients would be reduced

e) Build up effect can be fully utilized, reducing skin dose [26,27]

f) The vMP is the only patient specific material, thus possible reduction of costs, storage space, etc.

In the pre-clinical aspect of this study, we have shown that manual prepositioning to within ± 2° and ±2 mm in 6 DoF according to infrared information can be performed even by first time users. Prepositioning human subjects took no longer than the phantom skull. With little practice, manual prepositioning is possible in well under one minute, the remaining corrections by the HexaPOD take ≤ 20 seconds. Thus, high precision 6 DoF positioning was expected be reliably possible in less than 2 minutes on actual patients; although the time for the actual manual pre-positioning could not be measured consistently due to logistic reasons, the expected time frame was basically confirmed in the pilot patient phase, where the mean duration of patient entering the treatment room to start of CBCT1 was 4.5 ± 1.5 minutes. The entire treatment session could on average be completed within the allocated 15 minute timeslot (mean 15.03 ± 6.01 minutes).

Combined semi-robotic repositioning accuracy in the phantom study showed a mean deviation to planning CT of 0.2 ± 0.2 mm and 0.07 ± 0.08° over all translations (xyz) and rotations respectively, close to the minimal system inaccuracies of the IR/image fusion systems themselves. These extraordinary results could however not be transferred to the clinical setting on patients. One possible reason is that the vMP itself was not removed between the phantom repositionings as it was from the patients. However, the repositioning of the vMP itself has been shown to be in the order of 0.1 mm on subjects[28] and is thus believed to be of lesser influence. The main reasons for this discrepancy must be the influence of tension in the repositioning process, which seems to remain an issue even with use of vacuum technology. Possibly, optimization of mouthpiece impression material and vMP casting will further improve these results in the future. Nonetheless, the clinical repositioning accuracy results shown in Table 4 and Table 5 still compare favourably to all available intracranial inter- and intra-fraction data attained by volume imaging of sorts (Additional file 1, Table S1).

Comparing these data to invasive frames is no easy matter. In general however, the mechanical accuracy of invasive frames is quite often overestimated and not necessarily submillimetric as exhaustively shown already by Maciunas et al. in 1994 [24]. A more recent and clinical paper comparing stereotactic invasive frame-based to image guided radiosurgery using kV imaging showed image guidance to be superior to reliance on stereotactic coordinates, possibly caused by mechanical inaccuracy and flex of the stereotactic frame[12].

We are not aware of pre existing results using the described method; van Santfort et al. however used the same vMP in comparing a BrainLab Mask system with and without this vMP using stereoscopic fluoroscopy imaging [6]. The best results were obtained with the vMP, quite similar to the inter- and intra-fraction results of this study (Additional file 1, Table S1). The authors conclude that fixation according to vMP alone is inferior to the combined method by comparing their data to historic vMP-based data. However such comparisons between the mV-portal and IGRT eras must be viewed with caution.

Some similarities of this method are shared with a University of Florida groups system [29,30] who also used an infrared reference frame reproducibly attached to a (non-vacuum) mouthpiece. However, they rely on a thermoplastic mask for positioning and fixation thus precluding a direct comparison with data presented here. Another group around Wiersma et al. recently described a very similar concept except without rigid fixation during treatment [31]. However, fixating the patient with a mechanical arm during treatment has virtually no drawbacks, eliminates the possibility of intra-fraction movement and thus the need for online position-tracking or correction.

Mechanical arms of sorts combined with a vMP have also been described previously, but, this was in essence a frame based system, requiring bilateral hydraulic-mechanical arms to remain rigidly attached to the vMP throughout the entire treatment series [32]. Although positioning flexibility was given, the hydraulic-mechanical arms could not reliably retain their full rigidity over a protracted treatment series spanning up to two months.

The drawbacks of the presented method are not yet obvious. Possibly, repositioning edentulous patients will pose problems, although both inter- as well as intra-fraction results of the one edentulous patient (patient 4) did not differ significantly from the dentate patients (p = 0.29 and p = 0.1 respectively) in the pilot study. These data however need to be viewed with caution due to the low numbers. To the authors knowledge, there is to date no published data comparing vMP positioning between edentulous and dentate patients.

Also, one might criticize that the system will be restricted to few institutions equipped with infrared cameras, CBCT and a 6 DoF couch; however, orthogonal fluoroscopy systems as in the Novalis system [33] or possibly even orthogonal megavoltage portal images could also be used instead of CBCT. The method would however need to be analysed to this respect as the lack of true volume imaging may limit the attainable precision due to out of plane rotations [34]. With practice, the head can be manually positioned to <2° and <2 mm under IR-guidance quickly (Table 2), thus, at least theoretically, the need for a 6DoF couch may be facultative as well, at least for treatments where small rotational inaccuracy is acceptable. The infrared cameras in the treatment room are however indispensible for this method. If the planning CT room lacks IR- cameras, an additional CBCT and further IR-based corrections prior to initial treatment would likely be necessary to attain submillimetric agreement with the planning CT position at first treatment (Figure 1). Considering the low dose applied by a cranial CBCT (0.9-1.2 mGy) [35] the additional CBCTs add very little radiation exposure.

On a more cautions note, the next steps are software and hardware optimizations as well as a large scale clinical study, currently in preparation; we expect the results to improve with increasing experience and user-friendliness of hard and software; currently, the recognition of the described infrared frame is not a clinically released option of *iGuide *which was not specifically designed for this functionality, so storing the patient- and isocentre-specific infrared frame position relative to room coordinates still needs to be simplified and visualization of the required corrections should also be improved.

In addition, combining vacuum mouthpiece and infrared frame into one rigid cast would probably not only increase precision but also simplify, expedite and increase the reliability of the process.

Once more data and experience is gathered, we expect that daily 3D imaging using ionizing radiation could be reduced to a typical once-weekly rate for all but the highest precisional requirements or hypofractionated series, as the indirect infrared information allowed excellent repositioning accuracy (mean 3D vector:1.6 ± 0.8 mm). In this case safety margins of 2 mm would be required according to the van Herk formula. If image guided 6 DoF corrections are performed prior to each treatment, the safety margins, namely those for intra-fraction movement, are submillimetric.

## Conclusions

Infrared-based manual 6 DoF prepositioning with robotic 6D correction of remaining translations and rotations has been shown to be a fairly simple and effective method in a clinical setting as well. Although the hypothesized submillimetric accuracy was not reached in the clinical setting, these initial results compare favourably with the best repeat positioning systems available.

## Competing interests

JW, RS, and MG have received travel reimbursements from Elekta, Crawley UK or Medical Intelligence. MV was co-founder of Medical Intelligence and was with the company between 1995 and 2007. Medical Intelligence was bought by Elekta in 2005. Since 2007 he has had no financial relations whatsoever with either Elekta or Medical Intelligence. None of the other authors have actual or potential conflicts of interest.

## Authors' contributions

JW contributed to study conception, coordination, data acquisition and analysis, MG contributed to coordination, patient treatment and data acquisition, BP contributed to patient treatment and data analysis, OS contributed to coordination, patient treatment and treatment planning, MV was involved in study conception, MF treated patients and contributed to conception and organization, RS contributed to conception, treated patients, data analysis and drafted manuscript. All authors revised the manuscript critically and gave final approval.

## Supplementary Material

Additional file 1 **Table S1: Inter- and Intra-fraction errors as analysed in IGRT for various repeat-fixation systems**[36-39]. Click here for file
