# Supplementary material for: Semi-robotic 6 degree of freedom positioning for intracranial high precision radiotherapy; first phantom and clinical results
Source: Radiat Oncol. 2010 May 26;5:42. doi: 10.1186/1748-717X-5-42 (PMC2890022; doi:10.1186/1748-717X-5-42)
Supplement: Additional file 1 — Table S1: Inter- and Intra-fraction errors as analysed in IGRT for various repeat-fixation systems[36-39]. [file 1748-717X-5-42-S1.DOC]

**Table S1. Inter- and Intrafraction errors as analysed in IGRT for various repeat-fixation systems**

|  |  |  |  |  | **INTERFRACTION** | | | |  | **INTRAFRACTION** | | | |
| --- | --- | --- | --- | --- | --- | --- | --- | --- | --- | --- | --- | --- | --- |
|  |  |  |  |  | **Translations** | **Rotations** (rms°) | | |  | **Translations** | **Rotations** (rms°) | | |
| **Author** | **year** | **System** | **Imaging** | **n** (inter/intra) | **3d Vector** (mean, mm/SD) | **LR** | **SI** | **AP** |  | **3d Vector** (mean, mm/SD) | **LR** | **SI** | **AP** |
| van Santvoort [6] | 08 | Stereotact. Mask & vMP | X-ray | 519/203 | 1.7± 0.7 | 0.38 | 0.4 | 0.38 |  | 0.44 | 0.27 | 0.15 | 0.14 |
| van Santvoort [6] |  | Stereotact Mask & UJS |  | 616/192 | 2.1± 1.2 | 0.8 | 0.95 | 0.95 |  | 0.59 | 0.46 | 0.49 | 0.48 |
| Boda Heggemann[13] | 06 | Thermopl Mask | CBCT | 25/2 | 4.7± 1.7 | n.a. | n.a. | n.a. |  | 1.34± 1.4 | n.a. | n.a. | n.a. |
| Boda Heggemann[13 |  | Scotch cast Mask |  | 30/4 | 3.1± 1.5 | n.a. | n.a. | n.a. |  | 1.9± 0.74 | n.a. | n.a. | n.a. |
| Willner[36] | 97 | Stereotact. Mask & UJS | CT | 22 | 2.4± 1.3 | n.a. | n.a. | n.a. |  | n.a. | n.a. | n.a. | n.a. |
| Masi[37] | 08 | Thermopl Mask&Bite Block | CBCT | 96 | 2.9± 1.3 | n.a. | n.a. | n.a. |  | n.a. | n.a. | n.a. | n.a. |
| Masi[37] | 08 | Thermopl Mask |  | 35 | 3.2± 1.5 | n.a. | n.a. | n.a. |  | n.a. | n.a. | n.a. | n.a. |
| Guckenberger [8] | 07 | Scotch cast Mask | CBCT | 12 | 3.0± 1.7 | n.a. | n.a. | n.a. |  | n.a. | n.a. | n.a. | n.a. |
| Guckenberger [8] | 07 | Thermopl. Mask |  | 8 | 4.6± 2.1 | n.a. | n.a. | n.a. |  | n.a. | n.a. | n.a. | n.a. |
| Fuss[38] | 04 | Thermopl. Mask | CT | 55 | 1.59± 0.8 | 0.67± 0.7 | 0.61± 0.6 | 0.67± 0.6 |  | n.a. | n.a. | n.a. | n.a. |
| Salter [39] | 01 | Talon invasive frame | CT | 26 | 1.38 ± 0.5 | 0.41 ± 0.4 | 0.29 ± 0.2 | 0.18 ± 0.1 |  | n.a. | n.a. | n.a. | n.a. |
| own data |  | HeadFlex (vMP) | CBCT | 102/110 | 1.6 ± 0.8 | 0.26±0.3 | 0.28±0.3 | 0.44±0.4 |  | 0.6 ± 0.4 | 0.15±0.1 | 0.23± 0.2 | 0.31±0.4 |

n.a.= not available

rms= root mean square
